# Fundamentals of Nonparametric Statistical Tests for Dental Clinical Research

**DOI:** 10.3390/dj12100314

**Published:** 2024-09-29

**Authors:** Arturo Garrocho-Rangel, Saray Aranda-Romo, Rita Martínez-Martínez, Verónica Zavala-Alonso, Juan Carlos Flores-Arriaga, Amaury Pozos-Guillén

**Affiliations:** 1 Pediatric Dentistry Postgraduate Program, Faculty of Dentistry, University of San Luis Potosí, 2 Manuel Nava, Zona Universitaria, San Luis Potosi 78290, Mexico; arturo.garrocho@uaslp.mx (A.G.-R.); sarayaranda@fest.uaslp.mx (S.A.-R.); 2 Master of Dental Sciences Program, Faculty of Dentistry, University of San Luis Potosí, 2 Manuel Nava, Zona Universitaria, San Luis Potosi 78290, Mexico; rita.martinez@uaslp.mx (R.M.-M.); nveroza@fest.uaslp.mx (V.Z.-A.); carlos.flores@uaslp.mx (J.C.F.-A.)

**Keywords:** biostatistical analysis, nonparametric statistics, dental research, evidence-based dentistry

## Abstract

This article provides the foundation for employing nonparametric testing in dental clinical research. To make wise judgments in their research, investigators should learn more about the main nonparametric tests and their particular uses. Biostatistical analysis is essential in dental research; dental research frequently deviates from the assumptions that underpin traditional parametric statistics. Nonparametric statistics are useful for studies with small sample sizes, nominal- or ordinal-level data, and non-normally distributed variables. These statistical tests make no assumptions about the sampled population. Nonparametric tests are statistical methods based on signs and ranks. For dental research to be conducted effectively and accurately, statistical approaches must be applied correctly. Therefore, dental researchers must understand the many statistical methods at their disposal and know when to use them.

## 1. Introduction

Evidence-based dentistry (EBD) is the integration of patient choices, clinical skills, and the best available information [1]. EBD is widely related to clinical decision-making in everyday dental practice. Oral clinicians find and use the most valid information, along with knowledge, experience, and judgment, to address real-world clinical problems in an effort to improve patient oral healthcare [2,3].

To determine the best evidence for a given clinical question, scientific information or evidence must be methodically gathered and analyzed. This evidence arises from well-designed studies that use different methodological designs, including systematic reviews (meta-analyses), experimental studies (randomized/controlled, cross-over, split-mouth, or non-randomized trials), and observational studies (cohorts, cross-sectional, or case–control), among others [4]. After that, the gathered information and findings must be directly applied to clinical practice. In dentistry, translational research plays a critical role in connecting the knowledge gap between reliable scientific findings and practical application (“from bench to bedside”). In order to determine the effectiveness, efficiency, and safety of novel oral interventions, this method gradually advances through experimental human research that incorporates data from in vitro and/or animal investigations [1,5].

A universal technique for evaluating a conclusion’s validity from dental clinical studies is statistical analysis. Statistical data analysis is a crucial component of a dental study [4,6]. Dental researchers can make inferences from ambiguous information and give meaning to apparently meaningless sets of numbers or data through the use of statistical techniques. As a result, the process is a creative production that gives data life [4,7]. A myriad of dental articles have described the principles of statistical analysis in dentistry in order to know the different methods for analyzing numerical data [8,9]. These data are collected from clinical, laboratory, or epidemiological studies that follow the methodological designs mentioned before.

However, the incorrect application of statistical techniques can lead to incorrect conclusions and inaccuracies, decreasing the article’s significance [10,11]. Numerous dentistry publications have been trying to recognize and minimize statistical mistakes. As a result, many articles have detected a wide range of these types of errors [12]. This fact has encouraged the editors to improve the quality of their publications by creating author and reviewer checklists or guidelines to help lower statistical mistakes [13]. One of the most common errors found in journals is the application of parametric statistical techniques to nonparametric data [7]. This is presumed to be because dental researchers have been trained mostly in parametric statistics, and many statistical software packages strongly support parametric statistical techniques [10].

Parametric tests are statistical tests that assume that the data are normally distributed and follows a symmetrical distribution. These tests typically have enough statistical power (they can detect a significant effect when one truly exists) and allow one to make inferences and predictions about a population based on sample data. So, parametric tests can obtain robust mathematical results. That is why these techniques are the most widely taught in universities. However, if the assumptions for the procedures are not met, it seems prudent, in the sense of robustness, to use nonparametric tests [10,11,14].

Nonparametric statistical tests (or “distribution-free tests”) are essential tools in dental clinical research, particularly when data do not meet the assumptions required for parametric tests [7,10,15]. These tests do not assume a specific distribution for the data and are often used when dealing with small sample sizes, ordinal data, or non-normally distributed data (severely skewed data) [6,10,13,16]. Research in the health sciences can greatly benefit from the application of nonparametric methodologies, despite the misunderstandings and little exposure to them [10,13]. Therefore, the present article aims to boost our understanding of nonparametric statistical analysis and its applications by providing actual and pertinent cases of the use of these techniques in dental research.

## 2. Why Nonparametric Tests?

Three main parametric assumptions—the normal distribution of the dependent variable, the sample size, and the degree of measurement—are consistently infringed by oral sciences researchers [6,11,13,16]. In this regard, in dental clinical research, data often do not follow the normal distribution due to various factors such as (i) small sample sizes, where many clinical studies involve limited participants (e.g., n < 25–30), leading to non-normal data distributions; (ii) ordinal data, where measures such as pain scales or satisfaction ratings are ordinal and not suitable for parametric tests; and (iii) outliers, where nonparametric tests are more robust against outliers that might skew parametric data distributions [10,11]. In terms of confirming the assumption of data normality, Shapiro–Wilk and Kolmogorov–Smirnov tests are sufficient to assess this property [6,11]. The main distinction between parametric and nonparametric statistical analysis is that the former employs original data values, while the latter uses just + or − signs or the rank of data sizes. Stated differently, nonparametric analysis is concerned more with the order of the data size than the actual value of the data [7,10,17]. According to Okoroiwu and Akwiwu [11], the main advantages of nonparametric tests are that (i) they require limited assumptions to be made about the format of the data when parametric techniques are not valid; (ii) it is less probable to reach incorrect conclusions because population assumptions are unnecessary; (iii) they can deal with unexpected outliers, which may be problematic with parametric methods; and (iv) they are very intuitive and do not require deep statistical knowledge. In contrast, the disadvantages of these tests are that (i) they may lack statistical power; (ii) they are oriented towards hypothesis testing rather than effect estimation; (iii) the necessary information is usually limited, so the results are more difficult to interpret; (iv) data information is not fully used; and (v) tied values may be problematic and adjustments are required.

## 3. Common Nonparametric Tests Employed in Dental Research

### 3.1. Pearson’s Chi-Square Test

The chi-square test compares proportions and tests the association between categorical variables. A random sample of data is used to assess how well the observed and expected outcomes fit together. It compares observed data frequencies to expected frequencies without correlation between variables. To ascertain whether the association is statistically significant, the test computes a chi-squared statistic, which is subsequently compared to a critical value [4,7,18,19]. A variant of the chi-square method is Fisher’s exact test, employed to ascertain whether two category variables significantly correlate with one another (2 × 2 contingency table). It is especially helpful when the sample size is small or the chi-square test’s assumptions are not met [4,7,20].

### 3.2. McNemar’s Test

McNemar’s test is a statistical test used for analyzing paired nominal data. The method is also applied to 2 × 2 contingency tables with dichotomous variables (e.g., “yes” or “no”, “female” or “male”, “healthy” or “sick”, etc.) of correlated or matched pairs of participants. In oral investigation, researchers often convert numerical data to dichotomous data before statistical analyses. This process, called dichotomization, makes data summarization more efficient and allows for a more simple interpretation of results. The McNemar test is the most appropriate tool for analyzing pre- and post-differences in dependent (related) samples [21].

### 3.3. Mann–Whitney U Test (Wilcoxon Rank Sum Test)

This test examines differences between medians, rather than means, from two separate groups where the dependent variable is either ordinal or continuous but not distributed normally [6]. It can be used in place of an unpaired *t*-test [15]. Two sets of raw data are combined and then scored based on an ordered classification. The data are ranked from lowest to highest, ignoring the group to which they belong. The sum of the ranks (not the original values) in each group and the respective medians are then statistically contrasted [4,7,15,17]. Nevertheless, the Mann–Whitney test lacks statistical power for relatively small samples. In fact, regardless of how much the groups differ, the Mann–Whitney test will always yield *p*-values greater than 0.05 if the total sample size is seven or less [22].

### 3.4. Wilcoxon Signed-Rank Test

The nonparametric analog of the *t*-test is the Wilcoxon signed-rank test, which may be used when the one-sample *t*-test assumptions are not met (small samples and the differences are not normally distributed). This more powerful method computes the difference in measurements between paired data or non-independent observations (e.g., before-and-after studies on the same participants or pair-sampled studies) [6]. It is a one-sample procedure for inferring the median difference in a matched pair setting, in which the relative magnitude of these differences is examined [17]. The procedure consists of converting the difference values to rank order and then computing the t statistic using the ranks (not the original observations) [4,7,17].

### 3.5. Kruskal–Wallis Test

The Kruskal–Wallis test is a nonparametric method that compares the medians of three or more independent groups to test whether samples originate from the same distribution [23]. It is used when the assumptions of the one-way ANOVA are not met, particularly the assumption of normality [4,24]. The method assigns ranks to the original raw values and then compares the sums of ranks to what would be expected if there were no differences among groups [7]. Post hoc median comparisons between pairs (e.g., Bonferroni’s correction, Dunn’s test, etc.) can be performed with the Mann–Whitney U test, adjusting the significance level [4,23,24].

### 3.6. Friedman Test

This rank test compares three or more related or matched groups, in which ranks replace the original data [11]. It is also useful when the data does not meet the assumptions required for a parametric test, such as the repeated measures ANOVA [23]. The scores of a single participant are ranked, and the total ranks are the same for each participant, automatically eliminating the differences among participants. Then, the sum of the ranks is calculated for each condition (or period), and the test statistic is computed. As with other nonparametric tests, tied values must be adjusted through averaging for rank computing [24]. The Friedman test also allows for multiple paired comparison post hoc procedures, which can be adjusted using the Bonferroni alpha level or through the Wilcoxon signed-rank test [23,25].

## 4. Examples of Application of Nonparametric Analysis in Dental Research

Table 1 [25,26,27,28,29,30,31,32,33,34,35,36,37,38] describes a variety of representative examples of recently published randomized controlled clinical trials (last 10 years) that applied nonparametric statistical tests in different areas of dental clinical research. In this table, several dental clinical studies are summarized, including aims, clinical field, methods (sample size, clinical procedures, outcome measurement methods, etc.), nonparametric analysis techniques, and main findings and conclusions. For this task, two experienced authors (AGR and APG) performed a PubMed search for recent articles (using the NCBI filter “Publication date: 10 years”). The following MeSH terms were used: “dental research”, “statistics, nonparametric”, and “clinical trials as topic”. These selected examples are presented intending to show the reader some applications of nonparametric statistical tests and their usefulness in dental clinical research.

## 5. Some Recommendations for the Appropriate Use of Nonparametric Tests

- Nonparametric tests are ideal for small sample sizes, but larger sample sizes increase test power.
- Ensure proper identification of the data type (ordinal, nominal, or continuous).
- Verify assumptions specific to each test.
- Collect data meticulously, considering potential biases.
- Use appropriate calibrated tools for measuring outcomes.
- Use statistical software for accurate calculations (e.g., SPSS, R, Jamovi, Excel, etc.).
- Interpret results in terms of clinical significance, not just statistical significance.

In summary, nonparametric tests are essential tools in dental research for analyzing ordinal and non-normal data. They provide robust and flexible methods for comparing groups and evaluating treatment outcomes, making them a crucial part of any clinical study. Understanding when and how to use these tests ensures robust and reliable results, aiding in better clinical decision-making and advancing dental research.

## 6. Limitations of Nonparametric Tests

It is important to consider the limits of nonparametric tests. When the underlying assumptions are met, these tests are less robust than parametric tests. Furthermore, nonparametric tests typically rely on weak assumptions about the distribution and/or population variance equality. Because they are continuous, they may not work for all data. Moreover, nonparametric tests assess the rankings rather than the original values and only consider order correlations among data, which may lead to a loss of information. Despite these drawbacks, nonparametric tests are the best option when the data do not comply with the conditions of parametric tests or when a distributional model for the data is not available [14].

## 7. Conclusions

For dental research to be conducted effectively and accurately, statistical approaches must be applied correctly. Therefore, it is critical that dental researchers understand the basic statistical methods at their disposal and know when to use them. Currently, nonparametric techniques are widely applicable to clinical research in dentistry. When it comes to nonparametric statistics, dental researchers need to be open to experimenting with and questioning these types of analytical techniques, since they are probably most appropriate for a significant number of current oral clinical trials.

## Figures and Tables

**Table 1:** Examples of published randomized controlled clinical trials that applied nonparametric statistical tests.

First Author and Year	Dentistry Area	Aim, Methodology, and Description of Statistical Analysis	Main Findings and Conclusions
Sahrmann et al., 2015 [26]	Periodontics	- To evaluate the impact of spraying 10% PVP–iodine simultaneously with subgingival rinse during subgingival instrumentation on the frequency and severity of oral bacteremia. - Subgingival instrumentation was conducted using either water or a PVP–iodine rinse. Prior to instrumentation, respondents applied the assigned solution by gargling for 1 min. The pockets were thereafter washed for one minute. The samples were subgingivally instrumented using liquid-cooled ultrasonic scalers (water/PVP–iodine) for 1 minute. - A blood sample was collected from the arm vein for quantitative microbiological examination. - In order to examine distinct variations in the prevalence of bacteremia among the groups, the McNemar test was conducted. Data on the number of bacteremia episodes and CFU were compared between groups using the Wilcoxon rank sum test.	- Following subgingival instrumentation with simultaneous PCP–iodine rinsing, bacteremia was markedly decreased, with a considerably lower number of colony-forming units (CFUs) (*p* = 0.003). - Conclusion: this approach is highly recommended for the treatment of periodontitis in patients who are at a much increased risk of endocarditis or infection of endoprostheses.
Balasubramanian et al., 2019 [27]	Maxillofacial surgery (oral traumatology)	- To evaluate and contrast the surgical access and postoperative outcomes of two intra-oral incisions used to treat fractures of the mandibular body. - Sixty patients with fractures of the mandibular body were assigned at random to either the control group (routine vestibular incision) or the experimental group (crevicular incision with vertical release). - Parameters of interest: swelling, muscular rigidity, sensory loss, wound healing, and gingival recession. - Comparison of continuous variables between the groups and time points was performed using the Mann–Whitney U test and the Friedman test, respectively. - To compare the proportions between time points, the McNemar test was applied. The chi-square test was utilized for comparing the proportions among different groups. For pairwise comparisons, Dunn’s test with Bonferroni correction was employed.	- Crevicular incision demonstrated better surgical outcomes in the immediate postoperative phase. - The difference in mouth opening, swelling, and neurosensory impairment between the two groups was also significant (*p* < 0.05). - The crevicular incision is a highly suitable option for obtaining surgical access and fixation of mandibular body fractures, resulting in decreased postoperative pain and improved surgical recovery.
Naumova et al., 2019 [28]	Preventive dentistry	- To investigate in adults the release of F- and in saliva from toothbrushing in comparison with sodium fluoride and amine fluoride. - Each group brushed their teeth either with (1) fluoridated bioactive glass dentifrice (FBGD) alone or with FBGD and (2) sodium fluoride or (3) amine fluoride (AmF). - Saliva was collected in time intervals before, immediately after, and after 30, 60, and 120 min. after toothbrushing. Fluoride concentration was determined using a fluoride ion selective electrode. - Results were statistically evaluated with the Mann–Whitney U test for independent variables and the Wilcoxon signed test for related variables.	- The increase in fluoride in saliva was higher after the application of NaF or AmF compared to fluoridated bioactive glass (*p* < 0.05). - The bioavailability of fluoride lasted longer (up to two hours) after the application of fluoridated bioactive glass (*p* < 0.05). - Conclusion: toothbrushing with fluoride-containing bioactive glass dentifrices had beneficial effects on fluoride bioavailability.
Sado-Filho et al., 2019 [29]	Pediatric dentistry	- This trial aimed to evaluate the clinical efficacy of intranasal ketamine and midazolam for procedural sedation in young children. - Uncooperative children under the age of seven were randomly assigned to three groups, (KMIN) receiving intranasal ketamine and midazolam; (KMO) receiving oral ketamine and midazolam; or (MO) receiving oral midazolam. - The link between behavior and the three groups was verified using Kruskal–Wallis and chi-square tests with Bonferroni correction, specifically Pearson’s or Fisher’s exact test. The Friedman test was used to compare vital signs at baseline and during the sedation session.	- The success of the treatment (assessed dichotomously) was KMIN 50.0%, KMO 46.4%, and MO 32.1% (*p* = 0.360). Adverse events were present in 44.0% of cases and were not statistically different among groups (*p* = 0.462). - Conclusion: all three regimens provided similar, minimal adverse effects associated with moderate dental sedation.
Ferreira et al., 2020 [30]	Endodontics	- The objective of this study was to assess and compare the frequency and severity of postoperative pain and the intake of analgesics following root canal treatment with various root canal sealers. - Sixty single-rooted teeth with asymptomatic necrosis and apical periodontitis were randomly divided into three experimental groups—AH Plus, Endofill, or MTA Fillapex—using root canal sealers. - The patients were directed to document the level of pain perceived as none, mild, moderate, or severe. Perceived pain levels were assigned scores ranging from 1 to 4 after 24 h, 48 h, and 7 days. - An analysis was conducted using the chi-square test to examine variations in the occurrence of postoperative pain and the requirement for an analgesic. Analysis of pain intensity differences following therapy was conducted using the ordinal chi-square test.	- The study did not reveal any statistically significant differences in terms of the occurrence or severity of postoperative pain or the requirement for analgesic use at any time point (*p* > 0.05). - The administration of AH Plus, Endofill, and MTA Fillapex for the purpose of filling root canals yielded identical rates of postoperative pain and requirement for analgesic medication.
Jablonski-Momeni et al., 2020 [31]	Orthodontics	- The objective of this study is to evaluate the effectiveness of the self-assembling peptide P11-4 in promoting remineralization when mixed with fluorides, as opposed to application of fluoride varnish alone. - Orthodontic brackets were permanently attached to enamel samples exhibiting white spot lesions. - The samples were randomly assigned to three groups; group I did not receive any treatment, group II had a single application of fluoride varnish at a concentration of 22,600 ppm, and group III was treated with P11-4 after this application. - Remineralization was assessed at three time points, baseline, post-demineralization, and following storage in a remineralization solution for 7 and 30 days. - The statistical analysis employed nonparametric tests, namely the Kruskal–Wallis test and the Friedman test.	- Significant differences were observed in the median ΔF (fluorescence loss %) values among all groups at all investigation times (*p* < 0.00001). - In conclusion, the usage of P11-4 in combination with fluoride varnish showed improved results compared to using fluorides alone for the purpose of remineralizing enamel adjacent to brackets.
Thoma et al., 2020 [32]	Periodontics and implantology	- To evaluate the long-term clinical, radiographic, and profilometric results at implant locations previously treated with either a volume-stable collagen matrix (VCMX) or an autogenous subepithelial connective tissue graft (SCTG). - The single implant locations of 20 patients were randomly assigned to either VCMX or SCTG. Re-examinations were conducted on patients at 6 months (6M), 1 year (FU-1), and 3 years (FU-3). - Comparisons of medians were assessed between the treatment groups using the Mann–Whitney U test and within a treatment group using the Wilcoxon signed-rank test.	- The median buccal mucosal thickness decreased by −0.5 mm and by −0.75 mm (*p* = 0.047) between BL and FU-3 (intergroup *p* = 0.303). - A median reduction of −0.2 mm (*p* = 0.039) in buccal soft tissue profilometry was observed between BL and FU-3 for VCMX, and a drop of −0.1 mm (*p* = 0.020) for SCTG (intergroup *p* = 0.596). - Conclusion: implant sites previously grafted with VCMX or SCTG showed minimal changes in the contour of the peri-implant tissue and the thickness of the soft tissue.
Bardini et al., 2021 [33]	Endodontics	- The objective of this study was to evaluate the results of non-surgical primary and secondary root canal treatments using either a new bioactive sealer and the single-cone approach, or gutta-percha, zinc oxide-eugenol sealer, and warm vertical compaction. - Two groups of sixty-nine patients were randomly assigned to receive treatment utilizing either the single-cone approach with BioRootTM RCS (Septodont) (BIO group) or warm vertical compaction with gutta-percha and ZOE sealer (PCS group). - The dichotomous variables were presented as the count of cases and the corresponding percentage, while the qualitative ordinal variables were presented as the count of cases and the median value. - Different nonparametric k-sample tests (e.g., Kruskal–Wallis test) were applied to verify the difference between the medians.	- The likelihood of survival was comparable in both the BIO and PCS groups (*p* = 0.4074) and the BIOAP and PCSAP groups (*p* = 0.9114). - Statistically insignificant, the success rate in the BIO groups was greater than in other groups (*p* = 0.0735). - Conclusion: both approaches demonstrated consistent outcomes after 12 months.
Follak et al., 2021 [34]	Restorative dentistry	- A random assignment of 211 NCCLs was made to four experimental groups, Scotchbond Universal Adhesive—SBU (3M Oral Care) and Prime & Bond Elect—PB (Dentsply Sirona), specifically designed for endodontic and squamous epithelium conditions. - Resin composite restorations were performed utilizing the Filtek Z250 system manufactured by 3M Oral Care. - The restorations were evaluated either initially or subsequently after a 6-month interval. - Statistical tests: restorative failures among the experimental groups were analyzed using the Kruskal–Wallis and Mann–Whitney U nonparametric tests.	- Significant statistical differences were seen among groups in terms of failures (*p* = 0.000 for both Foreign Direct Investment and US Petroleum History System criteria). - In comparison to the other experimental groups, PB-SE exhibited a higher number of failures (*p* < 0.05). There was no statistically significant difference seen between any another pair of groups (*p* > 0.05). - Conclusions: The etch and rinse approach affected the clinical performance of Prime & Bond Elect. For this material, the self-etch approach generated lower criteria scores after a six-month follow-up. - There was no observed impact of the bonding technique on the clinical performance of Scotchbond Universal Adhesive.
De la Torre et al., 2022 [25]	Craniofacial pathology (temporomandibular dysfunction)	- The present study aimed to evaluate the enduring impacts of botulinum toxin type A (BoNT-A) on subjective pain, pain sensitivity, and muscle thickness among individuals with chronic myofascial temporomandibular disorder pain (MFP-TMD). - Fourteen female participants with persistent MFP were randomly assigned to undergo bilateral therapy with BoNT-A at varying doses (10U-25U for the temporalis muscle and 30U-75U for the masseter muscle). - The clinical assessments were self-perceived pain by VAS, pain sensitivity through PPT, and muscle thickness measuring ultrasonography. The follow-up period was up to 72 months. - The Friedman test, together with the Bonferroni test, were employed to compare nonparametric repeated measurements across the evaluation sites.	- VAS values exhibited a substantial decline during the course of the research (*p* < 0.05). Regarding PPT values, a statistically significant increase was observed when comparing baseline data with post-treatment follow-ups (*p* < 0.05). - However, there was a significant decrease in muscle thickness when comparing baseline values with the 1- and 3-month assessments. No differences were found when comparing baseline values with the 72-month follow-up period (*p* > 0.05). - Based on the findings, it can be concluded that a single injection of BoNT-A has enduring benefits in alleviating pain in individuals with persistent MFP-TMD, and the negative impacts on masticatory muscle thickness can be reversed.
Li H et al., 2022 [35]	Craniofacial pathology (migraine)	- The present pilot study aimed to examine the hemodynamic status of peripheral blood immune cells in individuals diagnosed with and without recurrent episodic or chronic migraine. - Grouped according to age, gender, and body mass index (BMI). - Continuous variables and comparisons between participants were performed with the Wilcoxon signed-rank test.	- Migraineurs had a markedly reduced proportion of non-classical monocytes (CD14+CD16++) in their bloodstream as compared to the control group. - Furthermore, migraineurs had a markedly reduced proportion of blood CD3+ CD4+ helper T cells and CD4+ CD25+ regulatory T cells in comparison to the control group. - More pronounced differences were seen in leukocyte surface markers between chronic migraine patients and their matched controls compared to episodic migraine patients and their matched controls. - Results: migraine was linked to disrupted balance of the peripheral immune system, and inflammation and autoimmune processes may contribute to its development.
Oppitz et al., 2024 Hongtao et al., 2022 [36]	Craniofacial pathology (bruxism treatment)	- To assess and compare the efficacy of a cost-effective, mixed occlusal splint (MOS) in comparison to a rigid splint. - A group of 43 adults diagnosed with sleep bruxism were randomly assigned to receive either rigid occlusal splints (ROS) or MOS. - Different features were assessed, such as masticatory muscle and temporomandibular joint function, pain severity (VAS), quality of life, indentations in the oral mucosa, anxiety and depression levels, duration of splint use, and splint wear. - Measurements were taken at three time points, baseline (T0), 6 months (T6), and 12 months (T12). Additionally, splint wear was assessed at T6 and T12. - The statistical methods used in this study included Student’s *t*-test, Mann–Whitney U test, nonparametric Friedman’s analysis of variance for paired samples and multiple comparisons, the chi-square test, the two-proportion z-test, the McNemar test, Cochran’s Q test, and the Wilcoxon test.	- Both groups saw a decline in trigeminal joint (TMJ) discomfort and pain severity, along with enhancements in quality-of-life scores as time progressed. - At time point 6, the MOS group had a greater incidence of splint wear compared to the ROS group (*p* = 0.023). - The rate of wear for the MOS was found to be greater than that of the rigid splint, although the other variables exhibited comparable trends. - In conclusion, the utilization of a mixed splint seems to be efficient in managing sleep bruxism.
Feizbakhsh et al., 2022 [37]	Orthodontics and oral microbiology	- To investigate the occurrence of bacteremia following orthodontic miniscrew placement. - A quasi-experimental study was conducted on 30 orthodontic patients (19 females and 11 males), with a mean age of 23.67 ± 4.87 years, in whom miniscrews were positioned. - Two blood samples were obtained for aerobic and anaerobic cultures before and 30–60 seconds after the appliance placement. These samples were incubated for five days in an automated blood culture machine. - Data analysis was performed using the McNemar test to compare the presence of aerobic and anaerobic bacteria before and after miniscrew placement.	- The blood samples of 29 patients were negative for the bacteria before and after miniscrew placement. - After miniscrew placement, a patient’s blood sample was positive for aerobic and anaerobic bacteria. However, bacteremia was negative in the initial (preplacement) blood samples. - The authors concluded that miniscrew placement in orthodontic patients was not associated with bacteremia.
Atteya et al., 2023 [38]	Dental caries prevention	- The present study conducted a randomized clinical trial to assess the remineralizing properties of self-assembling peptide (P11-4), nanosilver fluoride (NSF), and sodium fluoride (NaF) on white spot lesions (WSLs) specific to permanent teeth. - A total of sixty-six young people with white spot lesions (WSLs) on the inner surfaces of their permanent teeth and with ICDAS code 1 or 2 were randomly divided into three groups, P11-4, NSF, or NaF. - Data on ICDAS, Nyvad lesion activity ratings, and Diagnodent values were recorded at baseline and at 1-, 3-, 6-, and 12-month intervals. - Comparative analysis was conducted between groups using the chi-square test, and within groups using the McNemar test.	- The change in ICDAS scores varied significantly among the three groups after 3 and 6 months (*p* = 0.005) according to statistical analysis. - Lesion activity, as measured by Nyvad scores, exhibited notable variations among the three groups, with the P11-4 group having the highest proportion of inactive instances. - Compared to NaF varnish, P11-4 and NSF varnishes showed a non-significant reduction in ICDAS. - The use of P11-4 and NSF varnish resulted in a decrease in the ICDAS scores, caries activity, and Diagnodent readings of white spot lesions (WSLs) across permanent teeth. Nevertheless, the percentage change in ICDAS scores did not show a statistically significant difference compared to NaF.

## Data Availability

No new data were created in this study. Data sharing does not apply to this study.
